# The effect of zonal harmonics on dynamical structures in the circular restricted three-body problem near the secondary body

**DOI:** 10.1007/s10569-020-09983-3

**Published:** 2020-09-25

**Authors:** Luke Bury, Jay McMahon

**Affiliations:** 1 Office 422 Aerospace Engineering Sciences Building, 3775 Discovery Dr, Boulder, CO 80303 USA; 2 Office 461 Aerospace Engineering Sciences Building, 3775 Discovery Dr, Boulder, CO 80303 USA

**Keywords:** Zonal harmonics, Circular restricted three-body problem, Orbital perturbations, Periodic orbits

## Abstract

The circular restricted three-body model is widely used for astrodynamical studies in systems where two major bodies are present. However, this model relies on many simplifications, such as point-mass gravity and planar, circular orbits of the bodies, and limiting its accuracy. In an effort to achieve higher-fidelity results while maintaining the autonomous simplicity of the classic model, we employ zonal harmonic perturbations since they are symmetric about the *z*-axis, thus bearing no time-dependent terms. In this study, we focus on how these perturbations affect the dynamic environment near the secondary body in real systems. Concise, easily implementable equations for gravitational potential, particle motion, and modified Jacobi constant in the perturbed model are presented. These perturbations cause a change in the normalized mean motion, and two different formulations are addressed for assigning this new value. The shifting of collinear equilibrium points in many real systems due to $J_2$ of each body is reported, and we study how families of common periodic orbits—Lyapunov, vertical, and southern halo—shift and distort when $J_2$, $J_4$, and $J_6$ of the primary and $J_2$ of the secondary body are accounted for in the Jupiter–Europa and Saturn–Enceladus systems. It is found that these families of periodic orbits change shape, position, and energy, which can lead to dramatically different dynamical behavior in some cases. The primary focus is on moons of the outer planets, many of which have very small odd zonal harmonic terms, or no measured value at all, so while the developed equations are meant for any and all zonal harmonic terms, only even terms are considered in the simulations. Early utilization of this refined CR3BP model in mission design will result in a more smooth transition to full ephemeris model.

## Introduction

The circular restricted three-body problem (CR3BP) is a useful platform for understanding and designing trajectories in the presence of two large, perturbing bodies (Szebehely 1967; Koon et al. 2006). Widespread use of the CR3BP has given rise to the field of low-energy trajectory design, which can help minimize fuel usage and allow access to a wide variety of orbital geometries (Howell 1983; Koon et al. 2006; Parker et al. 2013; Vaquero Escribano 2013; Bosanac 2016; Restrepo 2018). However, popular solutions obtained with the classical CR3BP are sometimes not dynamically robust, particularly in sensitive regions. The CR3BP makes large assumptions such as the circular motion of bodies and point-mass gravity. The purpose of this paper is to provide the reader easily implementable tools that can increase the accuracy of the CR3BP dynamic model. This goal is accomplished by modifying the CR3BP to account for zonal harmonic perturbations. These are terms that describe non-spherical properties of a body that happen to be symmetric about the *z*-axis, such as $J_2$, which describes the difference between a body’s equatorial and polar radii. Because of their *z*-axis symmetry, these perturbations are autonomous in the CR3BP, making them ideal candidates for further study. In order to make these perturbations easily implementable, derivations of the CR3BP gravitational potential, equations of motion, and modified Jacobi constant which account for zonal harmonic terms are presented, and the effects of these perturbations on collinear equilibrium points and periodic orbits are studied.

Certain zonal harmonics in the CR3BP have previously been considered. One of the immediate effects of accounting for zonal harmonics is a change in the system potential, which can affect the positions of the Lagrange equilibrium points. For a number of three-body environments in the Solar system, Sharma and Rao (1974) study the locations of all five equilibrium points when $J_2$ of the primary body is accounted for. Markellos et al. (2004) also consider $J_2$ of the primary, but study homoclinic and heteroclinic connections between Lyapunov orbits in the Hill problem. Jain and Aggarwal (2015) study non-collinear equilibria accounting for $J_2$ of the primary, but also account for a dissipative Stokes drag force from the primary body. Carvalho et al. (2014) study the design of near-circular frozen orbits when considering the third-body perturbations of Jupiter along with $J_2$, $J_3$, and $C_{22}$ of Europa. Zotos (2015) categorizes a wide range of starting conditions in a three-body system as being either bounded, escaping, or collisional trajectories, and studies how these categorizations change when accounting for $J_2$ of the primary. Cinelli et al. (2015) studied the use of polynomial equations to account for zonal harmonics of Europa up to $J_4$ and a second-order Legendre polynomial expansion to account for Jupiter’s third-body perturbations in order to find sun-synchronous orbits about Europa. Singh and Umar (2017) study periodic orbits around $L_4$ and $L_5$ when both bodies are oblate in the ER3BP. Cinelli et al. (2019) looked at long-lifetime, high-inclination orbits about Europa when considering $J_2$ and $C_{22}$ of Europa via a double-averaged expression of disturbing potential.

There are a number of studies that look at the location and stability of the equilibrium points when $J_2$ of both the primary and secondary bodies are accounted for Sharma and Rao (1974), Bhatnagar and Hallan (1979), Elshaboury (1989), Arredondo et al. (2012). In addition to analyses of equilibrium points, some studies look at the effects of zonal harmonics on planar periodic orbits. Certain periodic orbits have been shown to exist when $J_2$ is taken into account for only the primary body (Sharma 1972a, b, c), for only the secondary body (Mittal et al. 2009), and for both (Abouelmagd et al. 2013). Although most work in this field looks specifically at the $J_2$ term, some studies also consider the effects of $J_4$. Abouelmagd et al. (2015a) verify the existence of equilibrium points, find their locations, and study their stability when both $J_2$ and $J_4$ of the primary and secondary bodies are accounted for. These authors also show that the $L_4$ and $L_5$ equilibrium points have planar periodic orbits when $J_2$ and $J_4$ of the primary body are accounted for Abouelmagd et al. (2015b).

This study aims to bridge results from the previous literature and simultaneously fill in gaps. This is accomplished by developing concise, easily implementable equations of motion which are able to account for any zonal harmonic perturbations due to both the primary and secondary bodies in the CR3BP. The normalized mean motion of a system is also affected by the added perturbations, and two formulations are addressed for determining this new value. Locations of collinear equilibria are studied under these dynamics, and the effects of these terms are studied near the secondary bodies in the Jupiter–Europa and Saturn–Enceladus systems. Lyapunov, vertical, and halo families of periodic orbits are studied near the $L_2$ points in these systems. The primary effects of the selected zonal harmonics on these families are a combination of an *x*-axis shift of the family, distortions in shape, and changes in specific energy at a given time period. In this study, only the effects of even zonal harmonic terms are analyzed because the main focus is on the outer planets, many of which have little or no measured value for odd zonal harmonic terms.

## Analytical expressions for the inclusion of zonal harmonics in the CR3BP

Closely following the procedure presented in Roy’s *Orbital Motion* (Roy 2005) for deriving the classical CR3BP equations of motion, this section presents a method for also expressing zonal harmonic perturbations from both the primary and secondary bodies in the CR3BP equations of motion, gravitational potential, and modified Jacobi constant. The derivation starts by including terms for $J_2$, $J_3$, and $J_4$ of each body, and works toward general, recursive forms. It is important to note that by including these terms which do not vary with time, it is assumed that the secondary body is orbiting in the equatorial plane of the primary and that the spin axis of each body is aligned. This is a reasonable assumption for many three-body systems. Europa and Enceladus, for example, are each inclined at less than 1$^{\circ }$ from their primary’s equatorial plane and are also tidally locked.

**Fig. 1:**
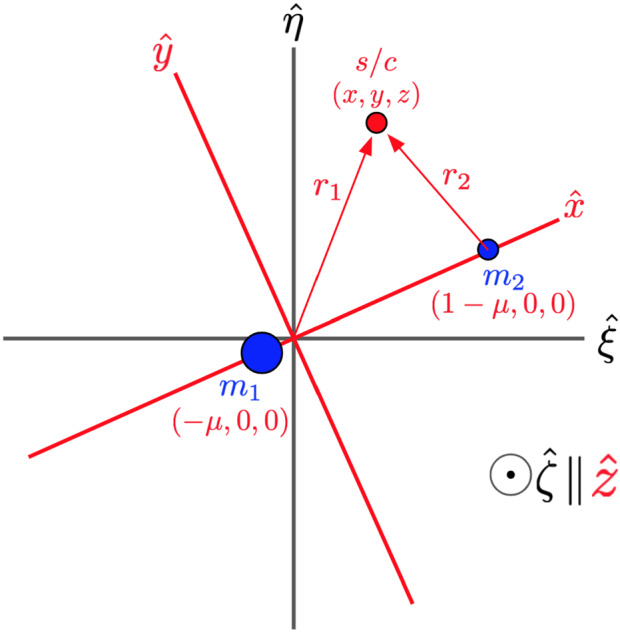
Layout of the circular restricted three-body problem, showing the relationship between the bodies and the inertial (black) and rotational (red) frames

To begin the derivation for the equations of motion, the normalization of the system and basic inertial frame are defined in Eqs. (1)–(2) where $M_1$ and $M_2$ are the masses of the primary and secondary body. The model is illustrated in Fig. 1. The velocities and positions of the bodies remain in the ${\hat{x}}$-${\hat{y}}$ plane, so the ${\hat{z}}$ and ${\hat{\zeta }}$ vectors remain constant and parallel.

*Normalization of the CR3BP system*1First, we define the coordinates of the spacecraft and each body in the inertial frame originating at the barycenter. In this formulation, the variables *x*, *y*, and *z* are reserved for coordinates in the rotating frame, while $\xi$, $\eta$, and $\zeta$ are the inertial frame equivalents.

2 $$\begin{aligned} s/c&: \{\xi , \eta , \zeta \},&r_1^2 = (\xi _1 - \xi )^2 + (\eta _1 - \eta )^2 + \zeta ^2,\nonumber \\ m_1&: \{\xi _1, \eta _1, \zeta _1\},&r_2^2 = (\xi _2 - \xi )^2 + (\eta _2 - \eta )^2 + \zeta ^2,\nonumber \\ m_2&: \{\xi _2, \eta _2, \zeta _2\}. \end{aligned}$$

From the recursive form of the equation for gravitational potential with zonal harmonics considered in Eq. (3) (Schaub and Junkins 2013), the accelerations due to both a primary and secondary body can be derived. The results of this derivation for the zonal harmonic terms of $J_2$-$J_4$ are expanded for clarity in Eqs. (4)–(7). To clarify some of the variables used in this section, with respect to the body being considered, *GM* refers to the gravitational parameter, *R* is the radius, *r* is the distance from the body to the spacecraft, $J_k$ is the *k*th zonal harmonic term, and $P_k(\frac{\zeta }{r})$ is the *k*th Legendre polynomial evaluated with $\frac{\zeta }{r}$.

3 $$\begin{aligned} V(r)&= \frac{GM}{r}\left[ 1 - \sum _{k=2}^{\infty }\left( \frac{R}{r}\right) ^k J_k P_k(\frac{\zeta }{r}) \right] . \end{aligned}$$

In Eq. (4), the equations of motion for the CR3BP derived without zonal harmonics are shown.

4 $$\begin{aligned} \ddot{\xi }_{3B}&= \dfrac{1 - \mu }{r_1^3}(\xi _1 - \xi ) + \dfrac{\mu }{r_2^3}(\xi _2 - \xi ),\nonumber \\ \ddot{\eta }_{3B}&= \dfrac{1 - \mu }{r_1^3}(\eta _1 - \eta ) + \dfrac{\mu }{r_2^3}(\eta _2 - \eta ),\nonumber \\ \ddot{\zeta }_{3B}&= -\dfrac{1 - \mu }{r_1^3}\zeta - \dfrac{\mu }{r_2^3}\zeta . \end{aligned}$$

Equations (5), (6), and (7) show the perturbing accelerations from $J_2$, $J_3$, and $J_4$, respectively. A notation system is introduced here where $J_{np}$ refers to $J_n$ of the primary, while $J_{ns}$ refers to $J_n$ of the secondary:

5 $$\begin{aligned} \ddot{\xi }_{J2}&= -\dfrac{3R_1^2 J_{2p}(1-\mu )(5\zeta ^2-r_1^2)}{2 r_1^7}(\xi _1-\xi ) - \dfrac{3 R_2^2 J_{2s}\mu (5\zeta ^2-r_2^2)}{2 r_2^7}(\xi _2-\xi ),\nonumber \\ \ddot{\eta }_{J2}&= -\dfrac{3R_1^2 J_{2p}(1-\mu )(5\zeta ^2-r_1^2)}{2 r_1^7}(\eta _1-\eta ) - \dfrac{3 R_2^2 J_{2s}\mu (5\zeta ^2-r_2^2)}{2 r_2^7}(\eta _2-\eta )\nonumber ,\\ \ddot{\zeta }_{J2}&= \dfrac{3R_1^2 J_{2p}\zeta (1-\mu )(5\zeta ^2-3r_1^2)}{2 r_1^7} + \dfrac{3 R_2^2 J_{2s}\zeta \mu (5\zeta ^2-3r_2^2)}{2 r_2^7}. \end{aligned}$$

6 $$\begin{aligned} \ddot{\xi }_{J3}&= -\dfrac{5R_1^3 J_{3p}\zeta (1-\mu )(7\zeta ^2-3r_1^2)}{2r_1^9}(\xi _1-\xi ) -\dfrac{5R_2^3 J_{3s}\zeta \mu (7\zeta ^2-3r_2^2)}{2r_2^9}(\xi _2-\xi ),\nonumber \\ \ddot{\eta }_{J3}&= -\dfrac{5R_1^3 J_{3p}\zeta (1-\mu )(7\zeta ^2-3r_1^2)}{2r_1^9}(\eta _1-\eta ) -\dfrac{5R_2^3 J_{3s}\zeta \mu (7\zeta ^2-3r_2^2)}{2r_2^9}(\eta _2-\eta )\nonumber ,\\ \ddot{\zeta }_{J3}&= \dfrac{R_1^3 J_{3p}(1-\mu )(3r_1^4-30r_1^2\zeta ^2+35\zeta ^4)}{2r_1^9} + \dfrac{R_2^3 J_{3s}\mu (3r_2^4-30r_2^2\zeta ^2+35\zeta ^4)}{2r_2^9}, \end{aligned}$$

7 $$\begin{aligned} \ddot{\xi }_{J4}&= -\dfrac{15R_1^4J_{4p}(1-\mu )(r_1^4-14r_1^2\zeta ^2+21\zeta ^4)}{8r_1^{11}}(\xi _1-\xi ) \nonumber \\&\quad -\dfrac{15R_2^4J_{4s}\mu (r_2^4-14r_2^2\zeta ^2+21\zeta ^4)}{8r_2^{11}}(\xi _2-\xi ),\nonumber \\ \ddot{\eta }_{J4}&= -\dfrac{15R_1^4J_{4p}(1-\mu )(r_1^4-14r_1^2\zeta ^2+21\zeta ^4)}{8r_1^{11}}(\eta _1-\eta ) - \nonumber \\ {}&\dfrac{15R_2^4J_{4s}\mu (r_2^4-14r_2^2\zeta ^2+21\zeta ^4)}{8r_2^{11}}(\eta _2-\eta ),\nonumber \\ \ddot{\zeta }_{J4}&= \dfrac{5R_1^4J_{4p}\zeta (1-\mu )(15r_1^4-70r_1^2\zeta ^2+63\zeta ^4)}{8r_1^{11}} \nonumber \\&+\dfrac{5R_2^4J_{4s}\zeta \mu (15r_2^4-70r_2^2\zeta ^2+63\zeta ^4)}{8r_2^{11}}. \end{aligned}$$

Now the expressions are condensed with a set of $\gamma \text { and } \beta$ variables. In this notation, $\gamma _{2p} \text { and } \gamma _{2s}$ correspond to the expressions for the effects of $J_{2p}$ and $J_{2s}$, respectively. The $\beta$ terms follow the same notations, but while $\gamma$ is used for x and y terms, $\beta$ is used for z terms.

8 $$\begin{aligned}&\gamma _{2p} =\dfrac{3R_1^2 J_{2p}(1-\mu )(5\zeta ^2-r_1^2)}{2 r_1^7},&\gamma _{2s} = \dfrac{3 R_2^2 J_{2s}\mu (5\zeta ^2-r_2^2)}{2 r_2^7},\nonumber \\&\beta _{2p} = \dfrac{3R_1^2 J_{2p}\zeta (1-\mu )(5\zeta ^2-3r_1^2)}{2 r_1^7},&\beta _{2s} = \dfrac{3 R_2^2 J_{2s}\zeta \mu (5\zeta ^2-3r_2^2)}{2 r_2^7},\nonumber \\&\gamma _{3p} = \dfrac{5R_1^3 J_{3p}\zeta (1-\mu )(7\zeta ^2-3r_1^2)}{2r_1^9},&\gamma _{3s} = \dfrac{5R_2^3 J_{3s}\zeta \mu (7\zeta ^2-3r_2^2)}{2r_2^9},\nonumber \\&\beta _{3p} = \dfrac{R_1^3 J_{3p}(1-\mu )(3r_1^4-30r_1^2\zeta ^2+35\zeta ^4)}{2r_1^9},&\beta _{3s} = \dfrac{R_2^3 J_{3s}\mu (3r_2^4-30r_2^2\zeta ^2+35\zeta ^4)}{2r_2^9},\nonumber \\&\gamma _{4p} = \dfrac{15R_1^4J_{4p}(1-\mu )(r_1^4-14r_1^2\zeta ^2+21\zeta ^4)}{8r_1^{11}},&\gamma _{4s} = \dfrac{15R_2^4J_{4s}\mu (r_2^4-14r_2^2\zeta ^2+21\zeta ^4)}{8r_2^{11}},\nonumber \\&\beta _{4p} = \dfrac{5R_1^4J_{4p}\zeta (1-\mu )(15r_1^4-70r_1^2\zeta ^2+63\zeta ^4)}{8r_1^{11}},&\beta _{4s} = \dfrac{5R_2^4J_{4s}\zeta \mu (15r_2^4-70r_2^2\zeta ^2+63\zeta ^4)}{8r_2^{11}}. \end{aligned}$$

Substituting into Eqs. (5), (6), and (7),

9 $$\begin{aligned} \ddot{\xi }_{J2}&= -\gamma _{2p}(\xi _1-\xi ) - \gamma _{2s}(\xi _2-\xi ),\nonumber \\ \ddot{\eta }_{J2}&= -\gamma _{2p}(\eta _1-\eta ) - \gamma _{2s}(\eta _2-\eta ),\nonumber \\ \ddot{\zeta }_{J2}&= \beta _{2p} + \beta _{2s}. \end{aligned}$$

10 $$\begin{aligned} \ddot{\xi }_{J3}&= -\gamma _{3p}(\xi _1-\xi ) - \gamma _{3s}(\xi _2-\xi ),\nonumber \\ \ddot{\eta }_{J3}&= -\gamma _{3p}(\eta _1-\eta ) - \gamma _{3s}(\eta _2-\eta ),\nonumber \\ \ddot{\zeta }_{J3}&= \beta _{3p} + \beta _{3s}. \end{aligned}$$

11 $$\begin{aligned} \ddot{\xi }_{J4}&= -\gamma _{4p}(\xi _1-\xi ) - \gamma _{4s}(\xi _2-\xi ),\nonumber \\ \ddot{\eta }_{J4}&= -\gamma _{4p}(\eta _1-\eta ) - \gamma _{4s}(\eta _2-\eta ),\nonumber \\ \ddot{\zeta }_{J4}&= \beta _{4p} + \beta _{4s}. \end{aligned}$$

With these substitutions, the inertial equations of motion are concise:

12 $$\begin{aligned} \ddot{\xi }&= \ddot{\xi }_{3B} + \ddot{\xi }_{J2} + \ddot{\xi }_{J3} + \ddot{\xi }_{J4},\nonumber \\ \ddot{\eta }&= \ddot{\eta }_{3B} + \ddot{\eta }_{J2} + \ddot{\eta }_{J3} + \ddot{\eta }_{J4},\nonumber \\ \ddot{\zeta }&= \ddot{\zeta }_{3B} + \ddot{\zeta }_{J2} + \ddot{\zeta }_{J3} + \ddot{\zeta }_{J4}, \end{aligned}$$

13 $$\begin{aligned} \ddot{\xi }&= \left( \dfrac{1-\mu }{r_1^3}-\gamma _{2p}-\gamma _{3p}-\gamma _{4p}\right) (\xi _1-\xi ) + \left( \dfrac{\mu }{r_2^3}-\gamma _{2s}-\gamma _{3s}-\gamma _{4s}\right) (\xi _2-\xi ),\nonumber \\ \ddot{\eta }&= \left( \dfrac{1-\mu }{r_1^3}-\gamma _{2p}-\gamma _{3p}-\gamma _{4p}\right) (\eta _1-\eta ) + \left( \dfrac{\mu }{r_2^3}-\gamma _{2s}-\gamma _{3s}-\gamma _{4s}\right) (\eta _2-\eta ),\nonumber \\ \ddot{\zeta }&= -\left( \dfrac{1-\mu }{r_1^3} + \dfrac{\mu }{r_2^3}\right) \zeta +\beta _{2p}+\beta _{3p}+\beta _{4p}+\beta _{2s} + \beta _{3s} + \beta _{4s}. \end{aligned}$$

To relate the inertial and rotating frames, we start by presenting relevant vectors in the rotating frame, which can all be seen in Fig. 1:

14 $$\begin{aligned} s/c&: \{x, y, z\},&r_1^2 = (x_1-x)^2 + y^2 + z^2,\nonumber \\ m_1&: \{x_1, 0, 0\} = \{-\mu , 0, 0\},&r_2^2 = (x_2-x)^2 + y^2 + z^2,\nonumber \\ m_2&: \{x_2, 0, 0\} = \{1-\mu , 0, 0\}. \end{aligned}$$

We now describe the inertial coordinates in terms of the rotating frame, which has rotated at an angle of *nt*, where *n* is the mean motion of the system:

15 $$\begin{aligned}&\xi = x \cos nt - y \sin nt,&\xi _1 = x_1 \cos nt,&\xi _2 = x_2 \cos nt,\nonumber \\&\eta = x \sin nt + y \cos nt,&\eta _1 = x_1 \sin nt,&\eta _2 = x_2 \sin nt,\nonumber \\&\zeta = z,&\zeta _1 = 0,&\zeta _2 = 0. \end{aligned}$$

Differentiating one has:

16 $$\begin{aligned} {\dot{\xi }}&= {\dot{x}} \cos nt - nx \sin nt - {\dot{y}} \sin nt - ny \cos nt,\nonumber \\ {\dot{\eta }}&= {\dot{x}} \sin nt + nx \cos nt + {\dot{y}} \cos nt - ny \sin nt,\nonumber \\ {\dot{\zeta }}&= {\dot{z}}. \end{aligned}$$

17 $$\begin{aligned} \ddot{\xi }&= (\ddot{x} - 2n{\dot{y}} - n^2x)\cos nt - (\ddot{y} + 2n{\dot{x}} - n^2y)\sin nt,\nonumber \\ \ddot{\eta }&= (\ddot{y} + 2n{\dot{x}} - n^2y)\cos nt + (\ddot{x} - 2n{\dot{y}} - n^2x) \sin nt,\nonumber \\ \ddot{\zeta }&= \ddot{z}. \end{aligned}$$

Now (15) is substituted into (13) and the sin and cos terms are collected. The $\ddot{\zeta }$ term is temporarily ignored due to its trivial conversion between inertial and rotational frames. See Eq. (17):

18 $$\begin{aligned} \ddot{\xi } =&\left( \dfrac{1-\mu }{r_1^3}-\gamma _{2p}-\gamma _{3p}-\gamma _{4p}\right) (x_1\cos nt - x\cos nt + y\sin nt) \nonumber \\&+\left( \dfrac{\mu }{r_2^3}-\gamma _{2s}-\gamma _{3s}-\gamma _{4s}\right) (x_2\cos nt - x\cos nt + y\sin nt),\nonumber \\ \ddot{\eta } =&\left( \dfrac{1-\mu }{r_1^3}-\gamma _{2p}-\gamma _{3p}-\gamma _{4p}\right) (x_1\sin nt - x\sin nt - y\cos nt) +\nonumber \\&+\left( \dfrac{\mu }{r_2^3}-\gamma _{2s}-\gamma _{3s}-\gamma _{4s}\right) (x_2\sin nt - x\sin nt - y\cos nt), \end{aligned}$$

19 $$\begin{aligned} \ddot{\xi }&= \left( (\dfrac{1-\mu }{r_1^3}-\cdots )(x_1- x) + (\dfrac{\mu }{r_2^3}-\cdots )(x_2 - x)\right) \cos nt\nonumber \\&\quad + \left( (\dfrac{1-\mu }{r_1^3}-\cdots ) + (\dfrac{\mu }{r_2^3}-\cdots )\right) y\sin nt,\nonumber \\ \ddot{\eta }&= \left( (\dfrac{1-\mu }{r_1^3}-\cdots )(x_1- x) + (\dfrac{\mu }{r_2^3}-\cdots )(x_2 - x)\right) \sin nt\nonumber \\&\quad - \left( (\dfrac{1-\mu }{r_1^3}-\cdots ) + (\dfrac{\mu }{r_2^3}-\cdots )\right) y\cos nt,\nonumber \\ \ddot{\zeta }&= -\left( \dfrac{1-\mu }{r_1^3} + \dfrac{\mu }{r_2^3}\right) z +\beta _{2p}+\beta _{3p}+\beta _{4p}+\beta _{2s} + \beta _{3s} + \beta _{4s}. \end{aligned}$$

Equating (19) with (17),

20 $$\begin{aligned}&(\ddot{x} - 2n{\dot{y}} - n^2x)\cos nt - (\ddot{y} + 2n{\dot{x}} - n^2y)\sin nt\nonumber \\&\quad = \left( \left( \dfrac{1-\mu }{r_1^3}-\cdots \right) (x_1- x) + \left( \dfrac{\mu }{r_2^3}-\cdots \right) (x_2 - x)\right) \cos nt \nonumber \\&\qquad + \left( \dfrac{1-\mu }{r_1^3}-\cdots +\dfrac{\mu }{r_2^3}-\cdots \right) y\sin nt, \end{aligned}$$

21 $$\begin{aligned}&(\ddot{y} + 2n{\dot{x}} - n^2y)\cos nt + (\ddot{x} - 2n{\dot{y}} - n^2x) \sin nt \nonumber \\&\quad = \left( \left( \dfrac{1-\mu }{r_1^3}-\cdots \right) (x_1- x) + \left( \dfrac{\mu }{r_2^3}-\cdots \right) (x_2 - x)\right) \sin nt + \nonumber \\&\qquad -\left( \dfrac{1-\mu }{r_1^3}-\cdots +\dfrac{\mu }{r_2^3}-\cdots \right) y\cos nt. \end{aligned}$$

By multiplying (20) by $\cos nt$ and (21) by $\sin nt$, adding, then separately multiplying (20) by $-\sin nt$ and (21) by $\cos nt$, and adding again, the following relation emerges^1^

22 $$\begin{aligned} \ddot{x} - 2n{\dot{y}} - n^2x&= \left( \dfrac{1-\mu }{r_1^3}-\gamma _{2p}-\gamma _{3p}-\gamma _{4p}\right) (x_1- x) + \left( \dfrac{\mu }{r_2^3}-\gamma _{2s}-\gamma _{3s}-\gamma _{4s}\right) (x_2 - x),\nonumber \\ \ddot{y} + 2n{\dot{x}} - n^2y&= -\left( \dfrac{1-\mu }{r_1^3} + \dfrac{\mu }{r_2^3}-\gamma _{2p}-\gamma _{3p}-\gamma _{4p}-\gamma _{2s}-\gamma _{3s}-\gamma _{4s}\right) y. \end{aligned}$$

By substituting in values for $x_1 \text { and } x_2$ from (14) and rearranging, we get the final equations of motion for the CR3BP model which includes $J_2$, $J_3$, and $J_4$ of each body:

23 $$\begin{aligned} \ddot{x}&= 2n{\dot{y}} + n^2x + \left( \dfrac{1-\mu }{r_1^3}-\gamma _{2p}-\gamma _{3p}-\gamma _{4p}\right) (-\mu - x) \nonumber \\&\quad + \left( \dfrac{\mu }{r_2^3}-\gamma _{2s}-\gamma _{3s}-\gamma _{4s}\right) (1-\mu - x),\nonumber \\ \ddot{y}&= - 2n{\dot{x}} + n^2y -\left( \dfrac{1-\mu }{r_1^3} + \dfrac{\mu }{r_2^3}-\gamma _{2p}-\gamma _{3p}-\gamma _{4p}-\gamma _{2s}-\gamma _{3s}-\gamma _{4s}\right) y,\nonumber \\ \ddot{z}&= -\left( \dfrac{1-\mu }{r_1^3} + \dfrac{\mu }{r_2^3}\right) z +\beta _{2p}+\beta _{3p}+\beta _{4p}+\beta _{2s} + \beta _{3s} + \beta _{4s}. \end{aligned}$$

### Recursive equations of motion, gravitational potential, and Jacobi constant

From (23), there is a clear pattern which allows the full equations to be written in recursive form.

24 $$\begin{aligned} \ddot{x}&= 2n{\dot{y}} + n^2x + \frac{\mathrm{d}V_p}{\mathrm{d}x} + \frac{\mathrm{d}V_s}{\mathrm{d}x},\nonumber \\ \ddot{y}&= -2n{\dot{x}} + n^2y + \frac{\mathrm{d}V_p}{\mathrm{d}y} + \frac{\mathrm{d}V_s}{\mathrm{d}y},\nonumber \\ \ddot{z}&= \frac{\mathrm{d}V_p}{\mathrm{d}z} + \frac{\mathrm{d}V_s}{\mathrm{d}z}, \end{aligned}$$

25 $$\begin{aligned}&\text {where}\nonumber \\ V_p&= \frac{1-\mu }{r_1}\left[ 1 - \sum _{k=2}^{\infty }\left( \frac{R_1}{r_1}\right) ^k J_k P_k(\frac{z}{r_1}) \right] ,\nonumber \\ V_s&= \frac{\mu }{r_2}\left[ 1 - \sum _{k=2}^{\infty }\left( \frac{R_2}{r_2}\right) ^k J_k P_k(\frac{z}{r_2}) \right] . \end{aligned}$$

With this form, the full gravitational potential for the CR3BP with zonal harmonics is clear:

26 $$\begin{aligned} U(r)&= \frac{1}{2}n^2\left( x^2 + y^2\right) + V_p + V_s, \end{aligned}$$

thus,

27 $$\begin{aligned} \ddot{x}&= 2n{\dot{y}} + \frac{\mathrm{d}U}{\mathrm{d}x},\nonumber \\ \ddot{y}&= -2n{\dot{x}} + \frac{\mathrm{d}U}{\mathrm{d}y}\nonumber ,\\ \ddot{z}&= \frac{dU}{dz}. \end{aligned}$$

Multiplying these three equations by ${\dot{x}},{\dot{y}},\text { and } {\dot{z}}$, respectively, and adding, results in:

28 $$\begin{aligned} {\dot{x}}\ddot{x} + {\dot{y}}\ddot{y} + {\dot{z}}\ddot{z} = \dfrac{\mathrm{d}U}{\mathrm{d}x}{\dot{x}} + \dfrac{\mathrm{d}U}{\mathrm{d}y}{\dot{y}} + \dfrac{\mathrm{d}U}{\mathrm{d}y}{\dot{y}}. \end{aligned}$$

Integrating this, an expression is found where *C* is a constant of integration and *V* is the magnitude of the particle’s velocity in the rotating frame. This *C* can be thought of as a modified Jacobi constant:

29 $$\begin{aligned}&{\dot{x}}^2 + {\dot{y}}^2+ {\dot{z}}^2 = 2U - C,\nonumber \\&V^2 = 2U - C, \end{aligned}$$

30 $$\begin{aligned}&C = -{\dot{x}}^2 - {\dot{y}}^2 - {\dot{z}}^2 + n^2\left( x^2 + y^2\right) + 2V_p + 2V_s. \end{aligned}$$

### Normalized mean motion in the zonal harmonics perturbed CR3BP

Let us denote the Keplerian mean motion as

31 $$\begin{aligned} n = \sqrt{\frac{GM}{a^3}}. \end{aligned}$$

Following the formula for Keplerian mean motion [Eq. (31)] and plugging in the standard normalization values [Eq. (1)], we see that in the classical formulation of the CR3BP, the normalized mean motion is equal to 1. However, when zonal harmonic perturbations are considered, this can no longer be assumed and a new formulation must be adopted. There are at least two approaches for coming up with this value. These approaches will be referred to as the “theory-based” and “ephemeris-based” methods, and each has advantages and disadvantages. The ephemeris-based method is used to generate the numerical results for this study. For clarity, a subscript ’n’ will be used to denote a normalized, dimensionless value (e.g., $T_{P_n} = 2\pi$, classically).

#### Theory-based method (example with $J_{2p}$ and $J_{2s}$)

For the theory-based method, the orbit of the primary–secondary system is treated as circular. The expression for mean motion is then determined by defining and equating the dynamical and kinematic expressions for $\ddot{\mathbf {r}}_{12}$ between the two gravitational bodies. To find the dynamic expression for $\ddot{\mathbf {r}}_{12}$, set up a system with two gravitational bodies and an inertial origin, define $\mathbf {r}_1$ and $\mathbf {r}_2$ as vectors from the origin to the respective bodies, find the expressions for $\ddot{\mathbf {r}}_1$ and $\ddot{\mathbf {r}}_2$ based on Newton’s laws and zonal harmonic potentials [Eq. (3)], and difference the two to find $\ddot{\mathbf {r}}_{12}$. A similar process has been seen in much of the literature regarding the three-body problem with zonal harmonics (Sharma and Rao 1974; Markellos et al. 2004; Jain and Aggarwal 2015; Zotos 2015; Singh and Umar 2017; Abouelmagd et al. 2013, 2015a, b).

In the unperturbed problem, this would show up as the recognizable:

32 $$\begin{aligned} \ddot{\mathbf {r}}_{12} = \frac{G(m_1+m_2)}{r_{12}^3}\mathbf {r}_{12}. \end{aligned}$$

However, when we include $J_{2p}$ and $J_{2s}$, we find the following relationship:

33 $$\begin{aligned} \ddot{\mathbf {r}}_{12} = \left[ \frac{G(m_1+m_2)}{r_{12}^3} + \frac{3G(m_1J_{2p}R_1^2 + m_2J_{2s}R_2^2)}{2r_{12}^5}\right] \mathbf {r}_{12}. \end{aligned}$$

Next, we want to solve for the kinematic expression for $\ddot{\mathbf {r}}_{12}$, which comes from a time derivative of the position vector:

34 $$\begin{aligned} \mathbf {r}_{12} =&\begin{bmatrix} a\cos {nt}\\ a\sin {nt}\\ 0 \end{bmatrix}, \end{aligned}$$

35 $$\begin{aligned} \ddot{\mathbf {r}}_{12} =&\begin{bmatrix} -an^2\cos {nt} \\ -an^2\sin {nt} \\ 0 \end{bmatrix}. \end{aligned}$$

Finally, by relating the dynamic and kinematic expressions for $\ddot{\mathbf {r}}_{12}$, an analytical expression for mean motion is obtained:

36 $$\begin{aligned} n^2 =&G\left[ \frac{m_1 + m_2}{a^3} + \frac{3(m_1J_{2p}R_1^2 + m_2J_{2s}R_2^2)}{2a^5}\right] , \end{aligned}$$

37 $$\begin{aligned} n_n^2 =&1 + \frac{3\left[ (1-\mu )J_{2p}R_1^2 + \mu J_{2s}R_2^2\right] }{2}. \end{aligned}$$

#### Ephemeris-based method

The key to this method is that no attention is paid to specific perturbations which are included in the model. Instead, a time-normalizing constant, $t_N$, is formulated to ensure that $G(m_1+m_2) = 1$. This $t_N$ can then be used to normalize the real, measured value of mean motion.

To start, we recognize that the units of *GM* are $\frac{\hbox {km}^3}{\hbox {s}^2}$. This relationship can then be exploited to find $t_N$:

38 $$\begin{aligned}&G(m_1+m_2) \frac{t_N^2}{r_N^3} = 1, \end{aligned}$$

39 $$\begin{aligned}&t_N = \sqrt{\frac{r_N^3}{G(m_1+m_2)}}. \end{aligned}$$

Using $t_N$ to normalize the measured value of mean motion, we arrive at the final expression, which in general does not equal 1.

40 $$\begin{aligned} n_n&= n\; t_N. \end{aligned}$$

#### Comparison of theory-based and ephemeris-based methods

Following the theory-based method, the calculated $n_n$ value is consistent with the exact model being used. However, the analytical process can be laborious as a different formulation is required for any combination of perturbations being considered. Further, there are still assumptions being made in this formulation that do not describe real-world behavior, such as the fact that the full-body problem is not being addressed (i.e., the effects of $J_{2p}$ on $J_{2s}$ and vice versa). Theoretically, it may be that the $n_n$ value calculated with the theory-based method converges toward the ephemeris-based method as perturbations are added.

An advantage to the ephemeris-based method is that its formulation is independent of the perturbations being included in the model, so the process always remains the same. Since the measured mean motion is a function of all the real perturbations, when we normalize this value, all the perturbations are still being represented. It is possible that solutions produced from this method may translate more easily into an ephemeris model, although this has not been investigated. Along with this, the ephemeris-based method may provide the most flexible way to use real data while still keeping a simple model for numerical studies.

The main drawback of the ephemeris method is that it can overcompensate for perturbations—the normalized mean motion value is based off of all real perturbations, but the simplified dynamical model being used in the study might include very few of them.

To compare the effects of the two methods of obtaining normalized mean motion, Tables 1 and 2 present the shifting of the collinear equilibrium points when the normalized mean motion value is changed according to the theory-based method (Table 1) and the ephemeris-based method (Table 2), but no perturbations are added to the dynamic model itself. The point of this is to isolate the effects of a change in normalized mean motion. For the theory-based case, the normalized mean motion is calculated via Eq. (37)—that is, $J_{2p}$ and $J_{2s}$ are accounted for.

The results show that an increase in $n_n$ acts to move the collinear points closer to the origin, and a lower $n_n$ moves the points further away. The magnitude of shifting is larger in the ephemeris-based case for all systems except Saturn–Enceladus. An important distinction to be aware of when comparing equilibria shifting due to changes in modeled potential versus changes in normalized mean motion is that the former is a change occurring within the frame, while the latter is a change of the frame itself, so we are observing the results of very different processes. In the remainder of the study, the ephemeris-based method for calculating $n_n$ is used to simplify the process when various zonal harmonic terms are being considered.

**Table 1:** Change in location along *x*-axis of collinear equilibrium points when the unperturbed CR3BP model is used, but the normalized mean motion is computed according to the theory-based method with $J_{2p}$ and $J_{2s}$

Primary	Secondary	$n_n$	$\Delta L1$ (km)	$\Delta L2$ (km)	$\Delta L3$ (km)
Earth	Moon	1.000000220	$-$ 0.0126	$-$ 0.0266	0.0564
Mars	Phobos	1.000192194	$-$ 0.4085	$-$ 0.3925	1.2011
Jupiter	Io	1.000302720	$-$ 26.8160	$-$ 29.9782	85.1027
Jupiter	Europa	1.000119599	$-$ 17.0232	$-$ 18.6702	53.5032
Jupiter	Ganymede	1.000047011	$-$ 10.4293	$-$ 11.9669	33.5457
Jupiter	Callisto	1.000015197	$-$ 5.9705	$-$ 6.7601	19.0735
Saturn	Enceladus	1.000731478	$-$ 39.9152	$-$ 37.4825	115.9652
Saturn	Titan	1.000027744	$-$ 6.8029	$-$ 8.3080	22.5981
Neptune	Triton	1.000012311	$-$ 0.8801	$-$ 1.0663	2.9116

**Table 2:** Change in location along *x*-axis of collinear equilibrium points when the unperturbed CR3BP model is used, but the normalized mean motion is computed according to the ephemeris-based method

Primary	Secondary	$n_n$	$\Delta L1$ (km)	$\Delta L2$ (km)	$\Delta L3$ (km)
Earth	Moon	0.999958544	2.3636	4.9951	$-$ 10.6119
Mars	Phobos	1.002596294	$-$ 7.4611	$-$ 4.0180	16.1935
Jupiter	Io	1.000545090	$-$ 48.3866	$-$ 53.8599	153.2085
Jupiter	Europa	1.000348441	$-$ 49.7160	$-$ 54.2540	155.8467
Jupiter	Ganymede	1.000157636	$-$ 34.9987	$-$ 40.0919	112.4728
Jupiter	Callisto	1.000178034	$-$ 70.0379	$-$ 79.0844	223.4205
Saturn	Enceladus	1.000375693	$-$ 20.0969	$-$ 19.6346	59.5783
Saturn	Titan	1.000210352	$-$ 51.6258	$-$ 62.9277	171.3120
Neptune	Triton	1.000180672	$-$ 12.9274	$-$ 15.6334	42.7221

## Effects on dynamic environment

Before studying the dynamical effects of zonal harmonic perturbations in the CR3BP, we present the system parameters that were chosen for this study. Tables 3 and 4 provide the chosen parameters relating to primary and secondary bodies, respectively. In Table 4, the semimajor axis, *a*, and the mean motion, *n*, are shown as these terms are necessary for system normalization.

When terms for zonal harmonics are included in the CR3BP, the gravitational potential of the system grows and the normalized mean motion changes. As a result, the collinear equilibrium points, $L_1$, $L_2$, and $L_3$, shift from their nominal locations. Knowing precisely where these equilibria are located is important for low-energy trajectory design, especially when looking to make use of dynamical systems theory to study periodic orbits and their manifolds. Table 5 provides the shifting seen in the collinear equilibria for real three-body systems when $J_2$ of both the primary and secondary is accounted for, and the ephemeris-based method is used to determine $n_n$ [Eq. (40)].

**Fig. 2:**
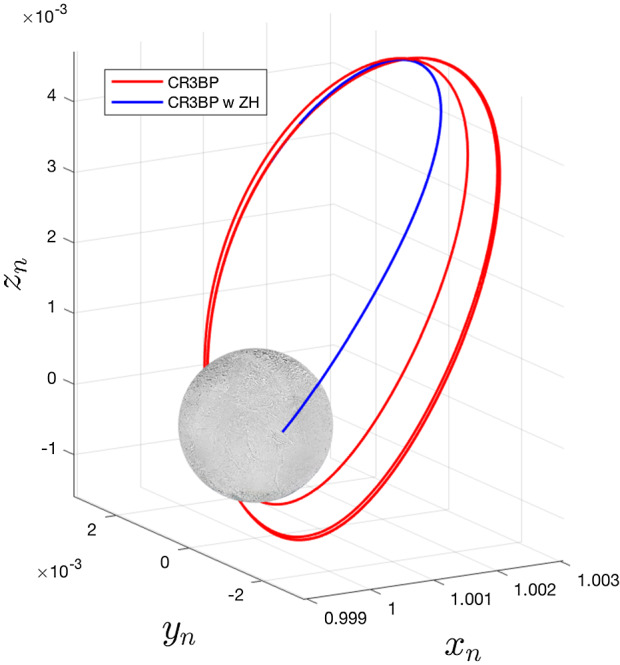
Comparing trajectories near Enceladus under the standard CR3BP dynamics (red) and a model also accounting for $J_{2p}$, $J_{4p}$, $J_{6p}$, and $J_{2s}$ (blue). The initial state for this trajectory is: $X_0 = [1.00185044467, 0.00304726277, 0.00256008094, 0.00315102743, -0.00170560198, 0.00623390101]$

As the system dynamics have changed and the Lagrange points have shifted, we can also expect trajectories to behave differently. One example of this in Fig. 2 shows a set of initial conditions corresponding to an unstable manifold from a northern halo orbit at Enceladus that, under standard CR3BP dynamics, goes on to impact the south pole at a near-tangent angle after several orbits. However, as we see in blue, when the same conditions are propagated with $J_{2p}$, $J_{4p}$, $J_{6p}$, and $J_{2s}$ accounted for, the trajectory quickly diverges from the nominal path and impacts Enceladus near the equator in less than one orbit. Clearly such differences could necessitate redesigning a targeted trajectory. To further study the effects of these zonal harmonic perturbations on trajectories, we examine shifts and distortions in common families of periodic orbits.

**Table 3:** Parameters of primary bodies used in this study

Primary	Radius (km)	Mass (kg)	$J_2$	$J_4$	$J_6$
Earth	6378	5.9742e24	0.0010826269	$-$ 0.0000016204	–
Mars	3390	6.39e23	0.00196045	–	–
Jupiter	69911	1.89819e27	0.0146956248	$-$ 0.0005913139	0.0000207751
Saturn	58232	5.683e26	0.0162906982	$-$ 0.0009343345	0.0000900350
Uranus	25362	8.681e25	0.00351068	$-$ 0.00003417	–
Neptune	24622	1.024e26	0.00340843	$-$ 0.0000334	–

**Table 4:** Parameters of secondary bodies used in this study

Secondary	Radius (km)	Mass (kg)	$\mu$ (mass ratio)	*a* (km)	*n* (rad/s)	$J_2$
Moon	1737.0	7.347e22	1.2148e-02	384748	2.66169953e-06	0.00020324
Phobos	11.3	1.0659e16	1.6681e-08	9376	2.28032986e-04	0.10058000
Io	1821.5	8.94e22	4.7095e-05	421800	4.11059240e-05	0.00081887
Europa	1560.8	4.799e22	2.5281e-05	671100	2.04782725e-05	0.00043340
Ganymede	2631.2	1.48e23	7.7963e-05	1070400	1.01644438e-05	0.00012544
Callisto	2410.3	1.08e23	5.6893e-05	1882700	4.35747966e-06	0.00002942
Enceladus	252.0	1.08022e20	1.9004e-07	237948	5.30733447e-05	0.00545979
Titan	2575.5	1.345e23	2.3661e-04	1221870	4.56080603e-06	0.00003341
Triton$^{2}$	1353.0	2.14e22	2.0894e-04	354759	1.23743166e-05	0.00043340

Estimating Triton’s $J_2$ to be equal to that of Europa (Correia 2009)

**Table 5:** Change in location along x-axis of collinear equilibrium points when $J_{2p}$ and $J_{2s}$ are added to the basic CR3BP and the ephemeris-based method is used to compute $n_n$

Primary	Secondary	$n_n$	$\Delta L1$ (km)	$\Delta L2$ (km)	$\Delta L3$ (km)
Earth	Moon	0.999958544	2.3875	5.0124	$-$ 10.6696
Mars	Phobos	1.002596294	$-$ 6.9122	$-$ 3.2581	14.9905
Jupiter	Io	1.000545090	$-$ 17.9892	$-$ 27.3898	68.0844
Jupiter	Europa	1.000348441	$-$ 30.8117	$-$ 37.4330	102.3327
Jupiter	Ganymede	1.000157636	$-$ 22.8899	$-$ 29.7683	78.9197
Jupiter	Callisto	1.000178034	$-$ 63.1953	$-$ 73.1792	204.3429
Saturn	Enceladus	1.000375693	18.8257	18.7456	$-$ 56.3595
Saturn	Titan	1.000210352	$-$ 43.1684	$-$ 56.2224	148.7009
Neptune	Triton	1.000180672	$-$ 11.8697	$-$ 14.7384	39.8091

## Effect on periodic orbits

**Fig. 3:**
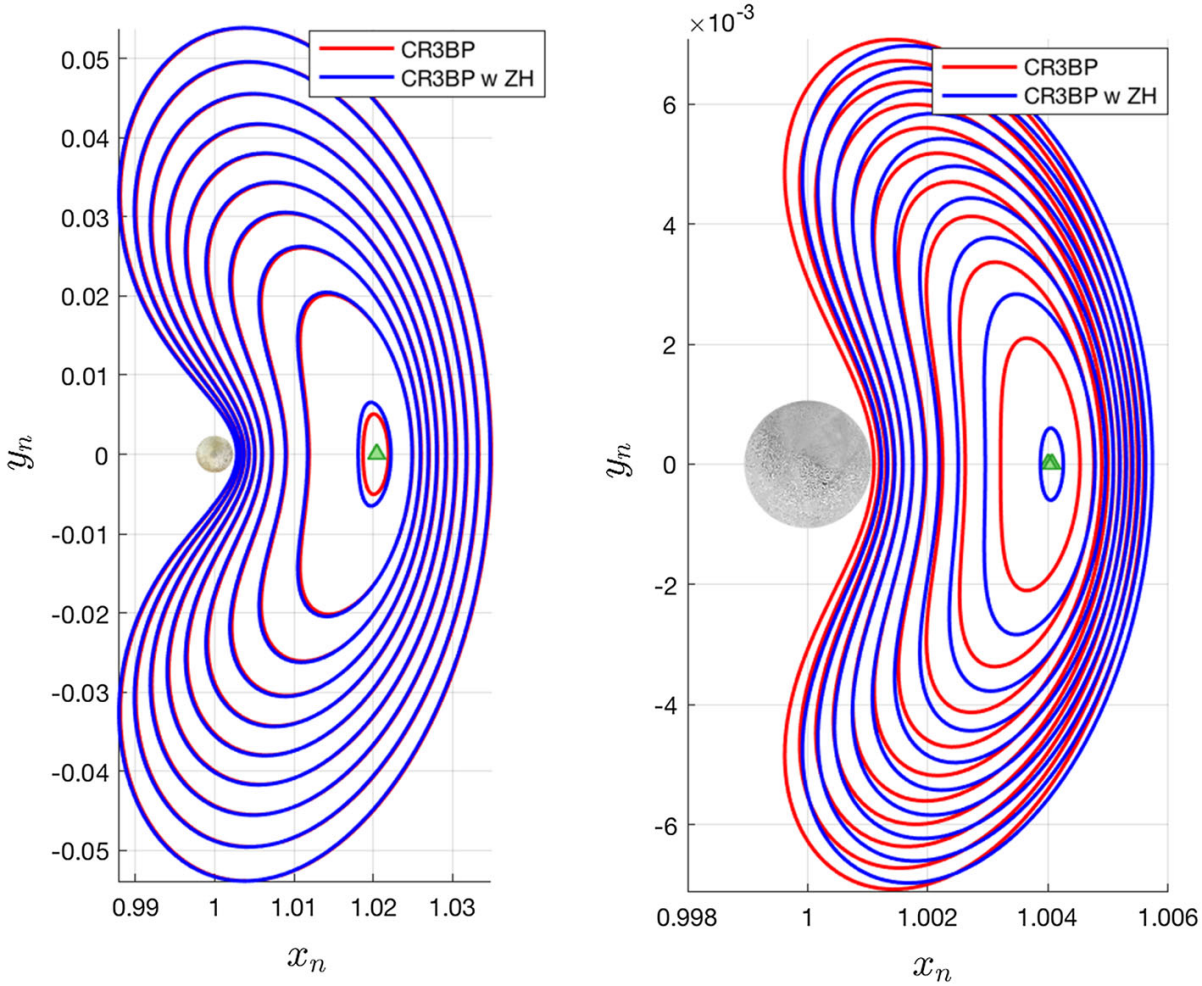
Lyapunov orbits computed with and without zonal harmonics accounted for, shown at Europa and Enceladus. In each system, 10 orbits are shown which have the same 10 time periods, spaced evenly from 3.09 to 5.51 at Europa, and 3.11 to 4.29 at Enceladus

**Fig. 4:**
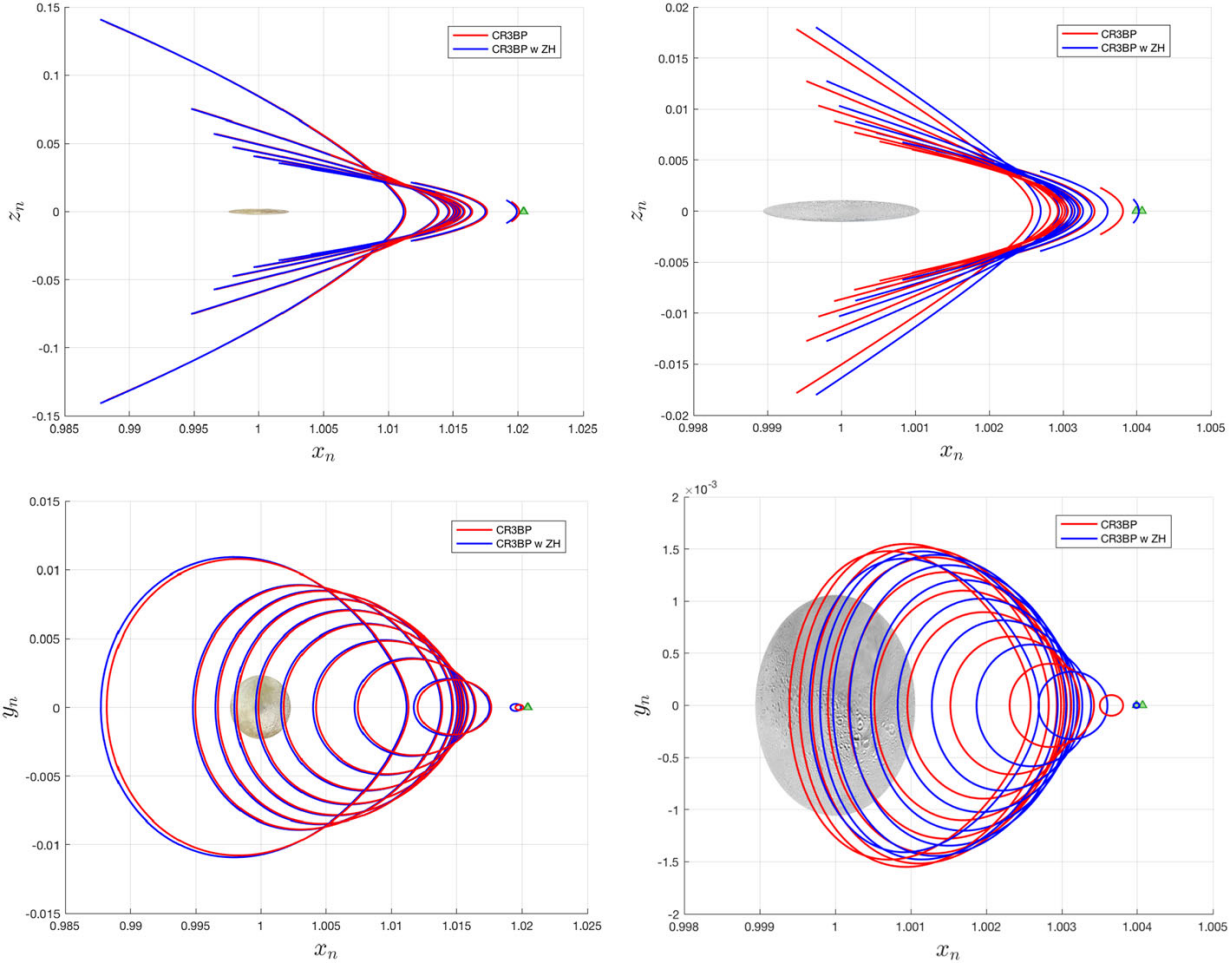
Vertical orbits computed with and without zonal harmonics accounted for, shown at Europa and Enceladus. In each system, 10 orbits are shown which have the same 10 time periods, spaced evenly from 3.22 to 6.18 at Europa, and 3.24 to 6.0 at Enceladus. Note—for the sake of more clearly showing the relative shape of the orbit families, axes shown are not equal in distance

Periodic orbits are inherently sensitive to initial conditions, so we can expect initial conditions computed in the classical CR3BP to not correspond to periodic orbits in the modified system. However, families can still exist, though they may shift and undergo changes in shape. Figures 3, 4, and 6 depict families of $L_2$ Lyapunov, vertical, and southern halo orbits at both Europa and Enceladus, with and without the inclusion of zonal harmonics. In each figure, 10 orbits from each model are shown. The 10 orbits correspond to matching time periods—thus, the largest blue and red orbits in each figure have the same period. The zonal harmonic terms included here are $J_{2p}$, $J_{4p}$, $J_{6p}$, and $J_{2s}$. It is clear that the effects of the chosen zonal harmonic terms have a more dramatic relative effect at Enceladus.

**Fig. 5:**
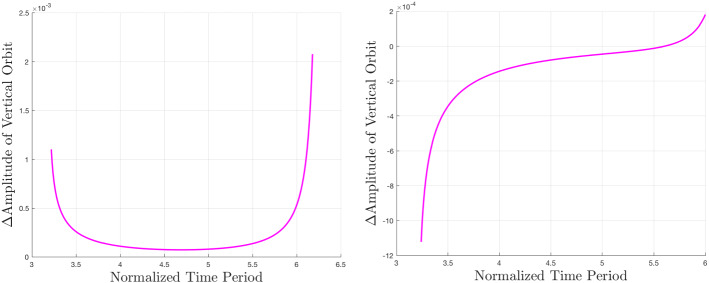
Changes in *z*-axis amplitude of $L_2$ vertical orbits as a function of time period at Jupiter–Europa (left) and Saturn–Enceladus (right)

**Fig. 6:**
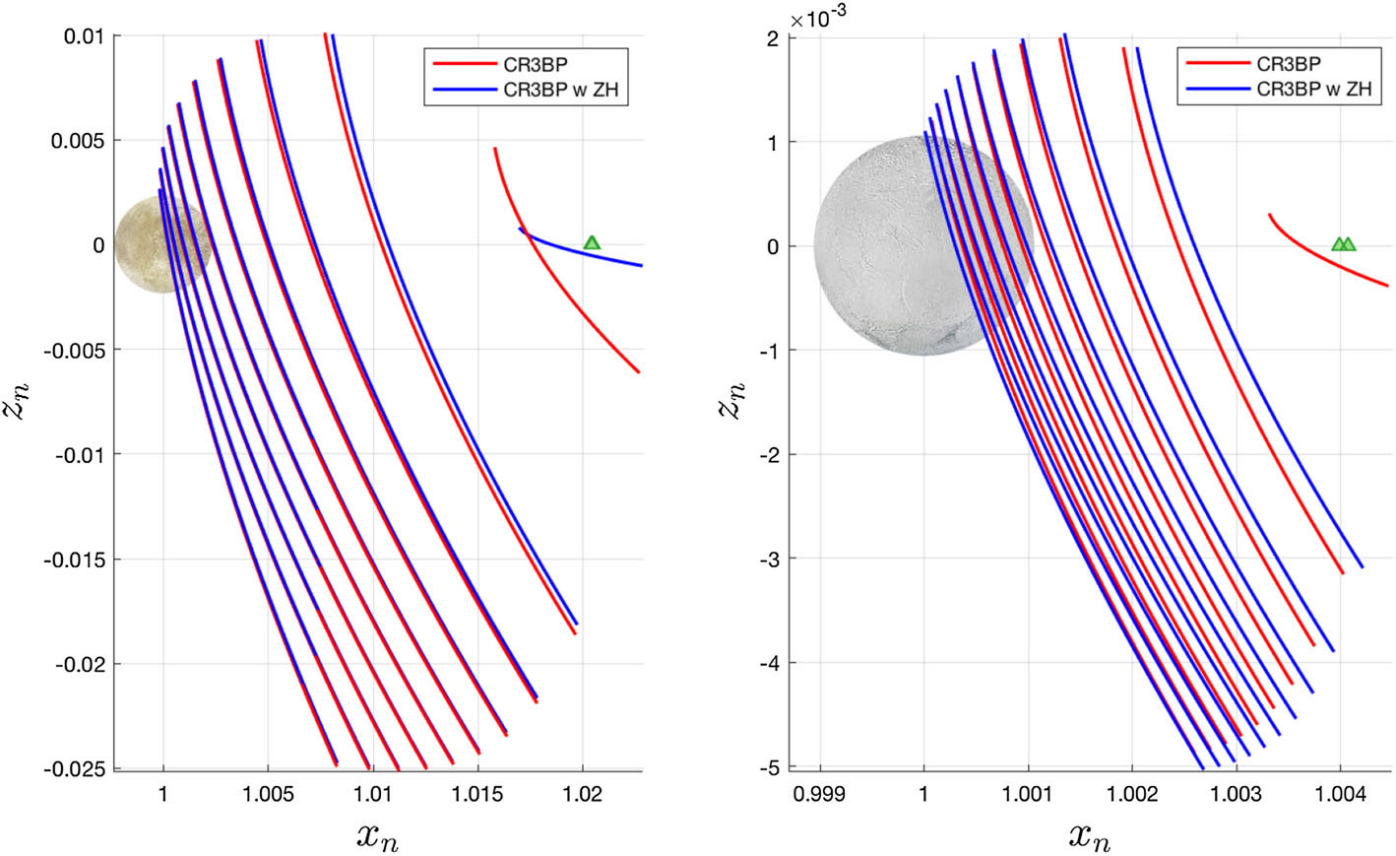
Southern Halo orbits computed with and without zonal harmonics accounted for, shown at Europa and Enceladus. In each system, 10 orbits are shown which have the same 10 time periods, space evenly from 1.81 to 3.114 at Europa, and 2.30 to 3.089 at Enceladus

A common theme among the newly computed families is an *x*-axis shift corresponding to the magnitude of the shift of the $L_2$ equilibrium point. Less intuitive is the change in shape exhibited by some families. For the case of Lyapunov orbits, we see a shortening of the family, or a reduction in *y*-axis amplitude at Enceladus, and the opposite at Europa. The magnitude of this effect is relatively greater at Enceladus. Changes to the vertical orbit family are not easily seen. The tall and skinny nature of vertical orbits makes them difficult for figures of this nature hoping to highlight differences between models, so the vertical orbits in Fig. 4 are shown with unequal axis units, hence the distorted shapes of Europa and Enceladus. Other than the apparent equilibrium point shift, it is not obvious what shape changes are occurring. One way this can be studied is by observing the change in *z*-axis amplitude between the two models for a given time period. This relationship is shown for Europa and Enceladus in Fig. 5. At Europa, we see that the effect of the zonal harmonics is to increase the amplitude of all vertical orbits in this range of time periods, but especially those that have particularly short or long periods. At Enceladus, we see that the zonal harmonics generate shorter vertical orbits at smaller periods and taller vertical orbits at larger periods. When comparing normalized distances between these two systems, it is important to remember that the normalized distance of $1\times 10^{-3}$ is equal to roughly 671 km at Europa and 238 km at Enceladus (Fig. 6).

Looking to the southern Halo families at Europa and Enceladus, a different trend emerges. Again by comparing orbits of the same time period, the included zonal harmonic terms decrease both the *y*- and *z*-axis ranges at Europa, while both are enlarged at Enceladus. An unexpected result is that unlike how vertical and Lyapunov families shift along the *x*-axis in the same direction as does the Lagrange point, the southern halo families shift in the opposite direction.

**Fig. 7:**
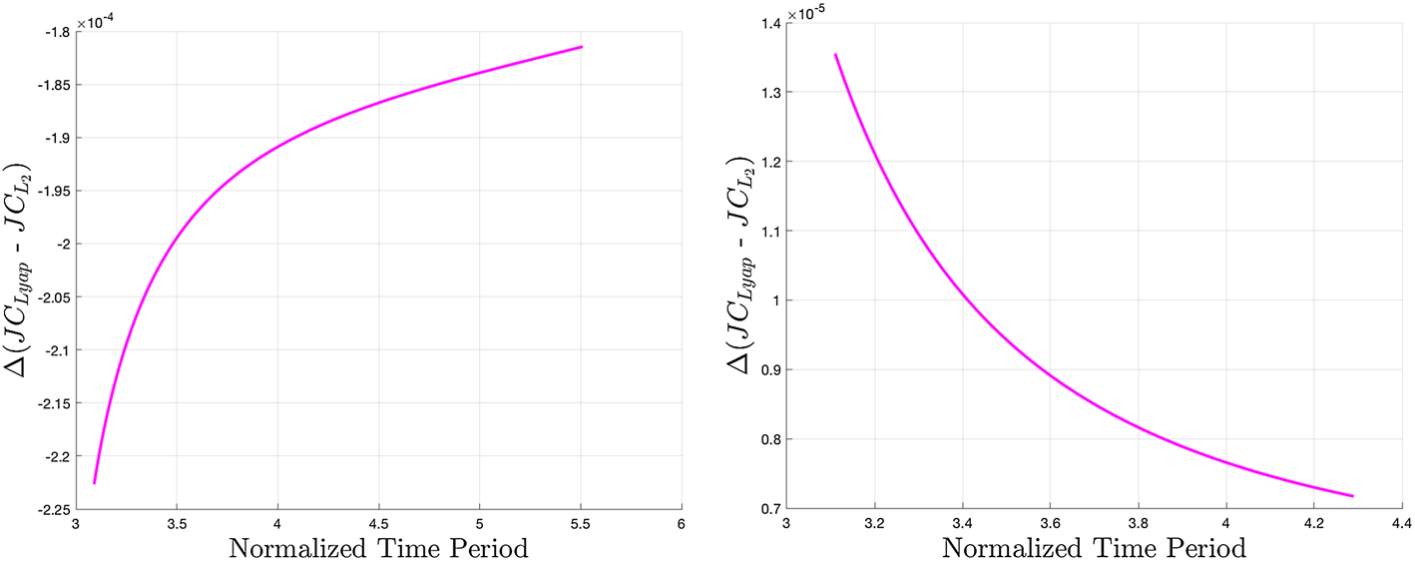
Approximate change in specific energy due to zonal harmonic perturbations for families of $L_2$ Lyapunov orbits at Jupiter–Europa (left) and Saturn–Enceladus (right)

**Fig. 8:**
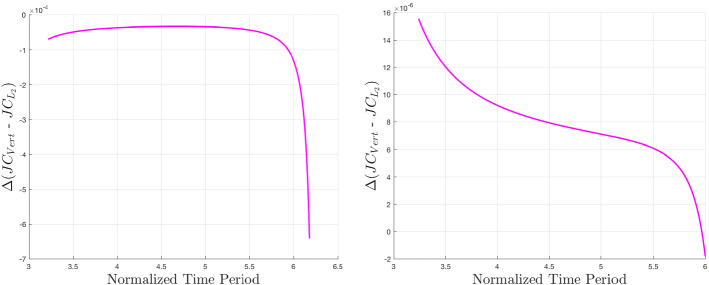
Approximate change in specific energy due to zonal harmonic perturbations for families of $L_2$ vertical orbits at Jupiter–Europa (left) and Saturn–Enceladus (right)

**Fig. 9:**
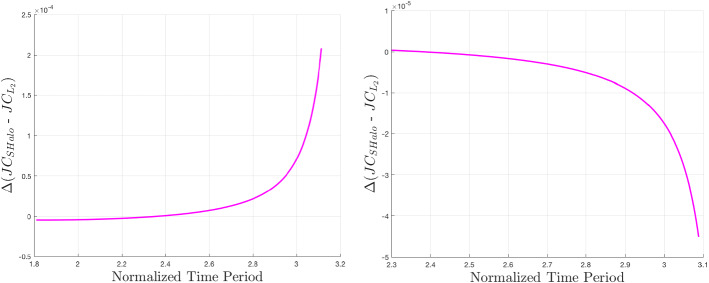
Approximate change in specific energy due to zonal harmonic perturbations for families of $L_2$ southern halo orbits at Jupiter–Europa (left) and Saturn–Enceladus (right)

To observe the effect of zonal harmonic perturbations on the energy of these periodic orbit families, we can study Figs. 7, 8, and 9 (Lyapunov, vertical, and southern halo, respectively). The *x*-axis values are time periods of periodic orbits since this value is monotonic across these families, making it a convenient parameter with which to scan a family. The *y*-axis of these figures is approximately a difference of specific energy between the two models. To determine the *y*-axis value, Jacobi constants are computed across a family in each model. Since the equations for Jacobi constant in the classical and perturbed models are fundamentally different, it is not valuable to compare specific values between the two. This can be addressed by differencing a Jacobi value with the Jacobi constant of $L_2$ in either model. This $\Delta JC$ between each periodic orbit and $L_2$ can be roughly translated as a specific energy, or $-v^2$ [Eq. (30)], which can be compared between models as long as the points of comparison are near each other in three-dimensional physical space. (Clearly from Figs. 3, 4, and 6, the families are relatively closer to each other at Europa than Enceladus, so larger errors may be present at the latter.) We subtract the $\Delta JC$ values computed for the classical CR3BP from those computed for the perturbed model. This is the relationship shown in the figures, and it describes approximate changes in energy to a family caused by the addition of zonal harmonics. At a specific time period on the *x*-axis, a negative value on the *y*-axis implies that the zonal harmonic perturbations have decreased the Jacobi constant, and therefore increased the specific energy. The figures reveal a complex relationship. For Lyapunov orbits with common time periods within the given bounds, the included zonal harmonics strictly raise the energy at Europa and decrease the energy at Enceladus. In each case, the magnitude of change decreases as time period is increased. For vertical orbits, energy strictly increases at Europa, but the sign of energy change is dependent on time period at Enceladus. Here, most orbits lose energy, but at the large end of the time period spectrum, orbits start to gain energy. Interestingly, we see from Fig. 5 that around $T_p=5.6$ at Enceladus, there is a point where zonal harmonics do not cause a change in *z*-axis amplitude, yet from Fig. 8, we see that at this point, specific energy is still changing. Looking at the southern halo results, the two systems have inverse results. At Europa, short-period halo orbits gain energy, but switch to losing energy at an increasing rate as time period grows. At Enceladus, short-period halo orbits lose energy, but quickly switch to gaining energy at an increasing rate.

## Conclusion

The equations of motion, gravitational potential, and modified Jacobi constant are all provided for the inclusion of zonal harmonics of each body in the circular restricted three-body problem. Specific equations of motion are provided for zonal harmonics through $J_4$, and recursive forms are provided so the reader can easily determine necessary equations for any choice of zonal harmonics.

The effect of $J_2$ of each body is studied by determining how the collinear equilibrium points shift along the *x*-axis for a variety of real moons in the Solar system. Determining these shifts is a non-trivial process that involves the understanding of two core pieces—the effects of the change in system potential and the effects of a change in normalized mean motion. By accounting for $J_2$ and any other zonal harmonics, the potential of the system is raised, and the result is that collinear equilibrium points shift away from the barycenter (when not accounting for changes in $n_n$). The effect that the normalized mean motion has on the collinear equilibrium points is dependent on its magnitude. If the perturbed normalized mean motion is larger than 1, its effect is to shift the collinear points toward the barycenter. However, if this value is less than 1, its effect is to shift the collinear points away from the barycenter. If the theory-based approach is used for calculating normalized mean motion, and only zonal harmonic terms are included in the model, then $n_n$ must be greater than 1. The ephemeris-based method is used in the generating of periodic orbits in this study.

To help study the real effects that these perturbations might have on mission design to the outer Solar system, families of Lyapunov, vertical, and southern halo periodic orbits are studied near the $L_2$ point in the Jupiter–Europa and Saturn–Enceladus systems. Families are located both in the classical model and in the model perturbed by $J_{2p}$, $J_{4p}$, $J_{6p}$, and $J_{2s}$. Odd zonal harmonic terms are ignored, since their values are very small or unmeasured at Jupiter and Saturn. These families are then compared against each other by viewing orbits with 10 common time periods, which make the relative shifting clear. It appears that $L_2$ Lyapunov and vertical orbits generally shift in the same direction as the $L_2$ equilibrium point when these zonal harmonic perturbations are considered, while the southern halo family appears to shift in the opposite direction as the equilibrium point. The families also undergo changes in shape and specific energy that are not consistent as a function of time period. The changes to shape can distort the periodic orbits by hundreds of kilometers.

The relationships found in this paper make it clear that the inclusion of zonal harmonic perturbations has the potential to greatly change early mission designs which are based on certain geometries or time periods, although the magnitudes of change caused by zonal harmonics are system-dependent. This result is useful for saving time in iterative mission design when solutions come to reckon with a full ephemeris model which accounts for such perturbations. The early inclusion of zonal harmonic terms can smooth the planning process and lead to a much more realistic solution.

## Appendix A

41 $$\begin{aligned}&\text {I: }\; A\cos {(t)} - B\sin {(t)} = C\cos {(t)} + D\sin {(t)} \end{aligned}$$

42 $$\begin{aligned}&\text {II: }\; B\cos {(t)} + A\sin {(t)} = C\sin {(t)} - D\cos {(t)} \end{aligned}$$

43 $$\begin{aligned}&\text {I} ^\prime : \text {I}\cdot \cos {(t)} = A\cos ^2{(t)} - B\sin {(t)}\cos {(t)} = C\cos ^2{(t)} + D\sin {(t)}\cos {(t)}\end{aligned}$$

44 $$\begin{aligned}&\text {II}^\prime : \text {II}\cdot \sin {(t)} = A\sin ^2{(t)} + B\sin {(t)}\cos {(t)} = C\sin ^2{(t)} - D\sin {(t)}\cos {(t)}\end{aligned}$$

45 $$\begin{aligned}&\text {I}^{\prime \prime }: \text {I}\cdot (-\sin {(t)}) = -A\sin {(t)}\cos {(t)} + B\sin ^2{(t)} = -C\sin {(t)}\cos {(t)} - D\sin ^2{(t)}\end{aligned}$$

46 $$\begin{aligned}&\text {II}^{\prime \prime }: \text {II}\cdot \cos {(t)} = A\sin {(t)}\cos {(t)} + B\cos ^2{(t)} = C\sin {(t)}\cos {(t)} - D\cos ^2{(t)} \end{aligned}$$

47 $$\begin{aligned}&\text {I}^\prime + \hbox {II}^\prime = A\left( \cos ^2{(t)} + \sin ^2{(t)}\right) = C\left( \cos ^2{(t)} + \sin ^2{(t)}\right) \end{aligned}$$

48 $$\begin{aligned}&A=C \end{aligned}$$

49 $$\begin{aligned}&\text {I}^{\prime \prime } + \hbox {II}^{\prime \prime } = B\left( \cos ^2{(t)} + \sin ^2{(t)}\right) = -D\left( \cos ^2{(t)} + \sin ^2{(t)}\right) \end{aligned}$$

50 $$\begin{aligned}&B=-D \end{aligned}$$

## Footnotes

1 Process detailed in “Appendix A”.
